# Supplementary material for: A Nucleus Accumbens Tac1 Neural Circuit Regulates Avoidance Responses to Aversive Stimuli
Source: Int J Mol Sci. 2023 Feb 22;24(5):4346. doi: 10.3390/ijms24054346 (PMC10001899; doi:10.3390/ijms24054346)
Supplement: Supplementary file 1 [file ijms-24-04346-s001.zip › ijms-2197836-caption of figures.pdf]

Supplementary Figure Legends

Figure S1. The activity of Tac1 neurons in the NAc lateral shell is not affected in response to aversive stimulus.

(A) Spikes elicited in NAc lateral shell Tac1 neurons in response to current injection. (B) Action potentials elicited after 200 pA current injection for 500 ms (ctrl, hindpaw saline injection: N = 4, n = 10; aversive, hindpaw formalin injection: N = 4, n = 10). Scale bar, 50 ms, 10 mV. n, cell number; N, animal number. Error bars represent SEM.

Figure S2. The activity of NAc Tac1 neurons does not affect time spent interacting with neural stimuli in mice.

(A) Schematic of strategies used to express AAV-DIO-mCherry, AAV-DIO-hM3D(Gq)-mCherry and AAV-DIO-hM4D(Gi)-mCherry in NAc medial shell of Tac1-Cre mice. (B) Schematic of avoidance assay (C: cotton). Control, hM3D(Gq) and hM4D(Gi) mice were i.p. injected by CNO (5 mg/kg). Heat maps display time spent in different regions of the chamber (warmer colors indicate more time). (C) Averaged time of control, hM4D(Gi) and hM3D(Gq) mice spent in different areas of the chamber (left: mCherry: 73.14 ± 8.110 s, hM4D(Gi): 85.33 ± 10.45 s, hM3D(Gq): 69.32 ± 11.12 s; centre: mCherry: 82.92 ± 5.090 s, hM4D(Gi): 76.40 ± 8.150 s, hM3D(Gq): 88.75 ± 4.619 s; cotton: mCherry: 143.9 ± 11.01 s, hM4D(Gi): 138.3 ± 6.816 s, hM3D(Gq): 141.9 ± 9.243 s. One-Way ANOVA test, left: $F(2, 19) = 1.282$, $P = 0.5428$; centre: $F(2, 19) = 1.157$, $P = 0.5789$; cotton: $F(2, 19) = 0.2486$, $P = 0.8921$). mCherry + CNO: N = 7; hM4D + CNO: N = 7; hM3D + CNO: N = 8. All data are means ± s.e.m. n.s., not significant.

Figure S3. hM3D(Gq) and hM4D(Gi) do not affect olfaction and locomotion in mice.

(A) Averaged latency time for mice to discovery food (latency, mCherry: 31.86 ± 6.002 s, hM4D(Gi): 30.43 ± 4.903 s, hM3D(Gq): 27.25 ± 3.315 s; One-way ANOVA test, $F(2,19) = 0.2552$, $P = 0.7774$). (B) Mean total distance traveled in the chamber (Travel distance, mCherry: 11.15 ± 1.304 m, hM4D(Gi): 12.04 ± 0.9996 m, hM3D(Gq): 12.71 ± 1.248 m; One-way ANOVA test, $F(2,19) = 0.4305$, $P = 0.6564$). mCherry + CNO: N = 7; hM4D + CNO: N = 7; hM3D + CNO: N = 8. N, animal number. All data are means ± s.e.m. n.s., not significant.

Figure S4. Anatomical Localization of NAc^{Tac1}-projecting neurons in the LH.

Localization of NAcTac1-projecting neurons across the Anterior-Posterior axis. LH, lateral hypothalamic area. Scale bar, 500 µm.

Figure S5. Total inhibitory charge transfer by optogenetic stimulation.

Average charge transfer of oIPSCs recorded. APV+CNQX: N = 3, n = 8; APV+CNQX+Bicuculline: N = 3, n = 6. N, animal number; n, cell number. All data are means ± s.e.m. ***$P < 0.001$.

Figure S6. Optogenetic inhibition of NAc^{Tac1}-LH induces real time place aversion in mice.

(A) Schematic showing of bilateral optogenetic stimulation of NAcTac1 Inputs to LH by NpHR. (B) Schematic of RTPA assay. RTPA: real-time place aversion. (C) Trajectory of the mice in the chamber. (D) Averaged percentage of time spent in the stimulation side (two-tailed paired t-test, $t14 = 5.671$, $P < 0.0001$). eYFP: N = 8; NpHR: N = 8. N, animal number. NAc msh, nucleus accumbens medial shell; LH, lateral hypothalamic area. All data are means ± s.e.m. ***$P < 0.001$.

Figure S7. The ratio of TVA and RV positive cells in the NAc medial shell

The percentage of TVA and RV positive cells in the total NAc medial population. N = 3. N, animal number. All data are means ± s.e.m.

Figure S8. Total excitatory charge transfer by optogenetic stimulation.

Average charge transfer of oEPSCs recorded. APV+ Bicuculline: N = 3, n = 8; APV+CNQX+Bicuculline: N = 3, n = 8. N, animal number; n, cell number. All data are means ± s.e.m. ***P < 0.001.

Figure S9. mPFC neurons send excitatory inputs to Tac1 neurons in the NAc medial shell.

(A) Schematic of strategies used to express AAV- CaMKIIα-ChR2-eYFP in Tac1-Cre: Ai9 mice. (B) Representative image of viral expression in the NAc medial shell. Scale bar, 10 µm. (C) The AMPA receptor antagonist CNQX totally inhibited oEPSCs in the NAc Tac1 neurons induced by optical stimulation. (D) Average amplitude of oEPSCs recorded (two-tailed paired t-test, $t_{14} = 11.12$, $P < 0.0001$). APV + Bicuculline: N = 3, n = 8; APV + CNQX + Bicuculline: N = 3, n = 8. N, animal number; n, cell number. All data are means ± s.e.m. ***P < 0.001.

Figure S10. Optogenetic inhibition of mPFC-NAc induces real time place aversion in mice.

(A) Schematic showing of bilateral optogenetic stimulation of mPFC Inputs to NAc by NpHR. (B) Schematic of RTPA assay. RTPA: real-time place aversion. (C) Trajectory of the mice in the chamber. (D) Averaged percentage of time spent in the stimulation side (two-tailed paired t-test, $t_{14} = 3.991$, $P = 0.0013$). eYFP: N = 8; NpHR: N = 8. N, animal number. NAc msh, nucleus accumbens medial shell; mPFC, medial prefrontal cortex. All data are means ± s.e.m. **P < 0.05.

Figure S11. Activation of the mPFC-NAc projections attenuated avoidance behaviors in response to aversive stimuli.

(A) Schematic of virus strategies. (B) Heat maps display time spent in different regions of the chamber (warmer colors indicate more time). (C) Averaged time of mCherry+hM4D(Gi) and hM3D(Gq)+hM4D(Gi) mice spent in different areas of the chamber (safe: mCherry+hM4D(Gi): 191.0 ± 7.859 s, hM3D(Gq)+hM4D(Gi): 165.3 ± 15.84 s; centre: mCherry+hM4D(Gi): 89.85 ± 11.03 s, hM3D(Gq)+hM4D(Gi): 105.6 ± 12.80 s; form: mCherry+hM4D(Gi): 17.69 ± 3.513 s, hM3D(Gq)+hM4D(Gi): 32.79 ± 4.885 s. two-tailed paired t-test, safe: $t_{12}= 1.456$, $P = 0.1709$; centre: $t_{12}= 0.9315$, $P = 0.3700$; form: $t_{12}= 2.510$, $P = 0.0274$). mCherry+hM4D(Gi): N = 7; hM3D(Gq)+hM4D(Gi): N = 7. All data are means ± s.e.m. *P < 0.05; n.s., not significant.
