# Supplementary figures and images for: A Nucleus Accumbens Tac1 Neural Circuit Regulates Avoidance Responses to Aversive Stimuli
Source: Int J Mol Sci. 2023 Feb 22;24(5):4346. doi: 10.3390/ijms24054346 (PMC10001899; doi:10.3390/ijms24054346)

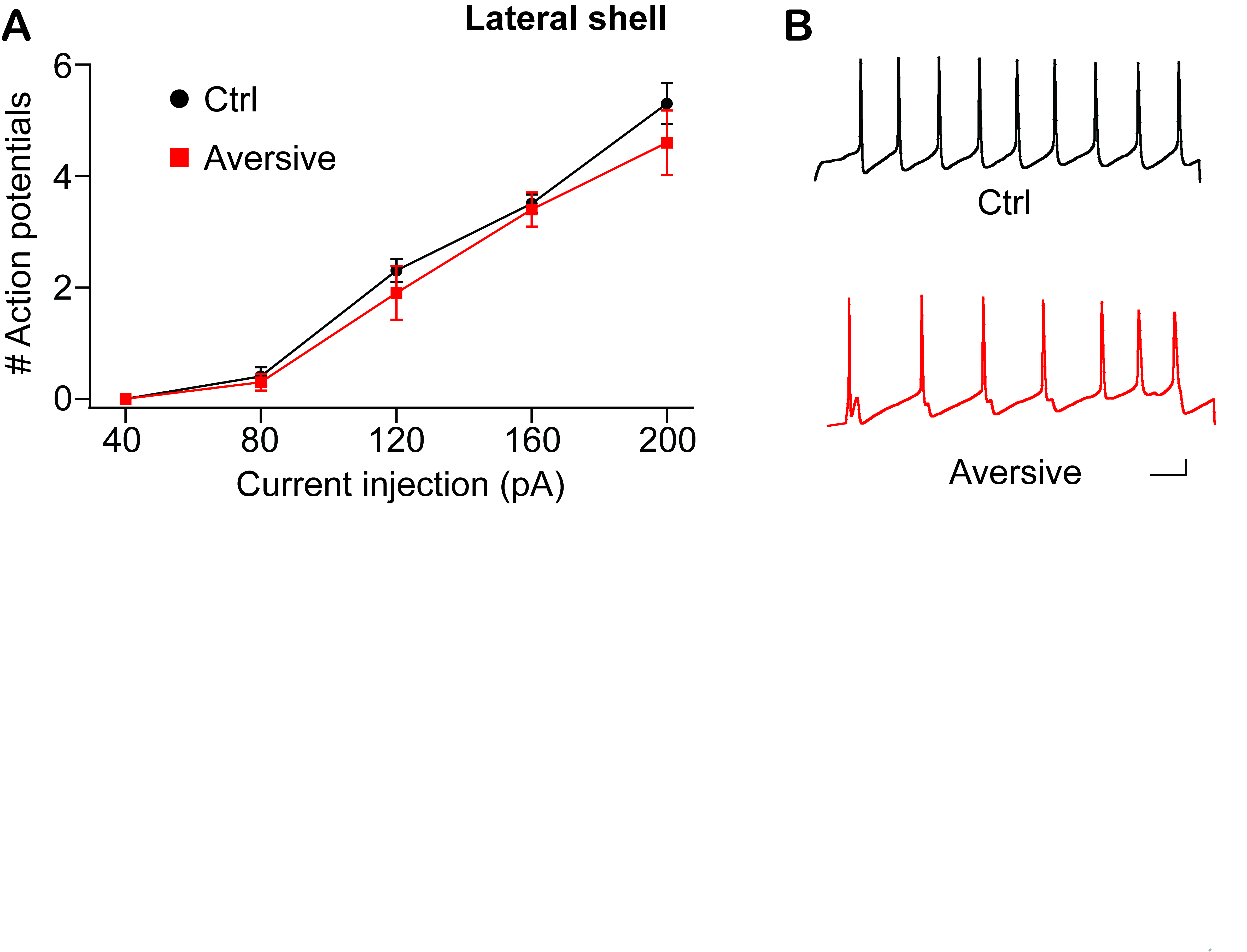

Supplement: Supplementary file 1 [file ijms-24-04346-s001.zip › Figure S1.tif]

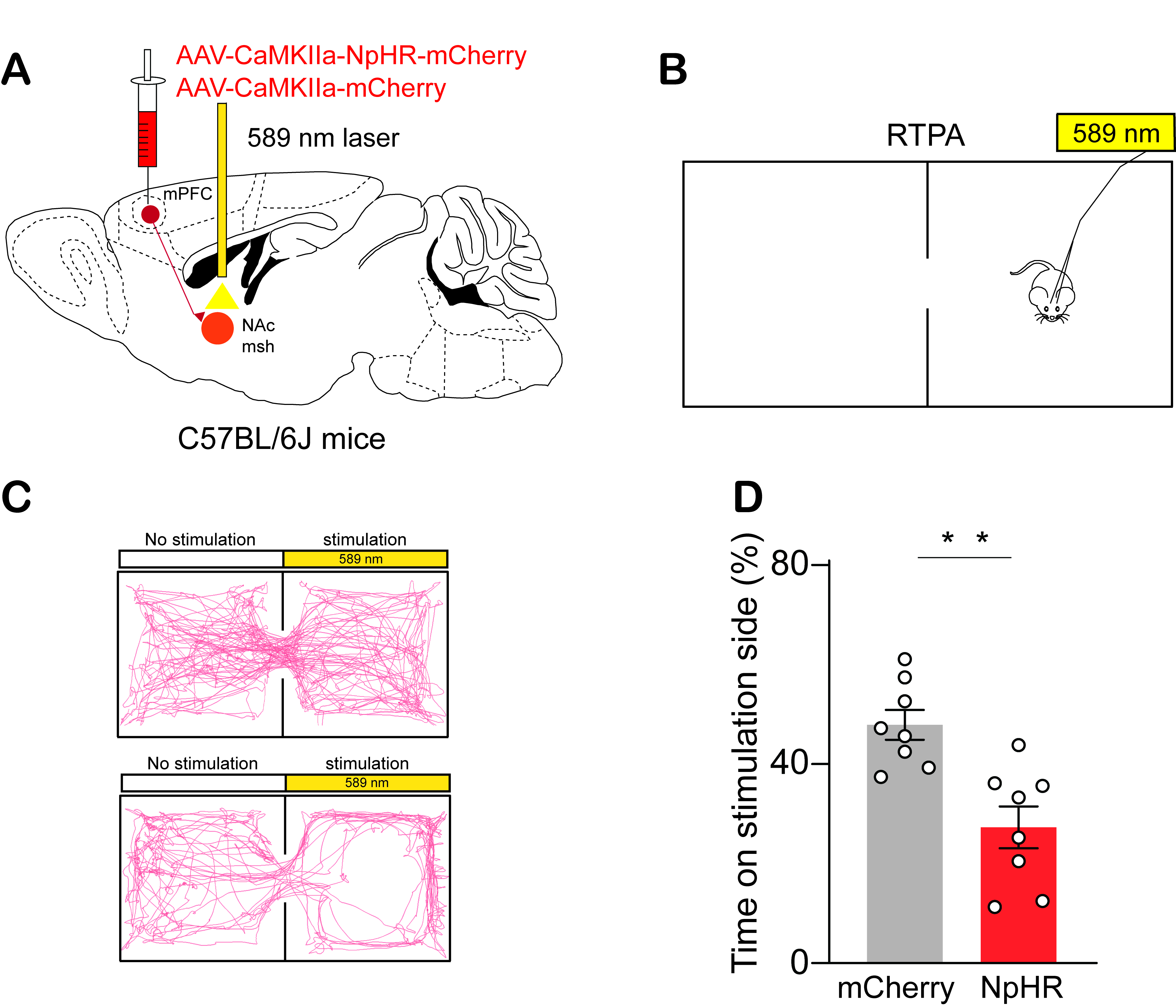

Supplement: Supplementary file 1 [file ijms-24-04346-s001.zip › Figure S10.tif]

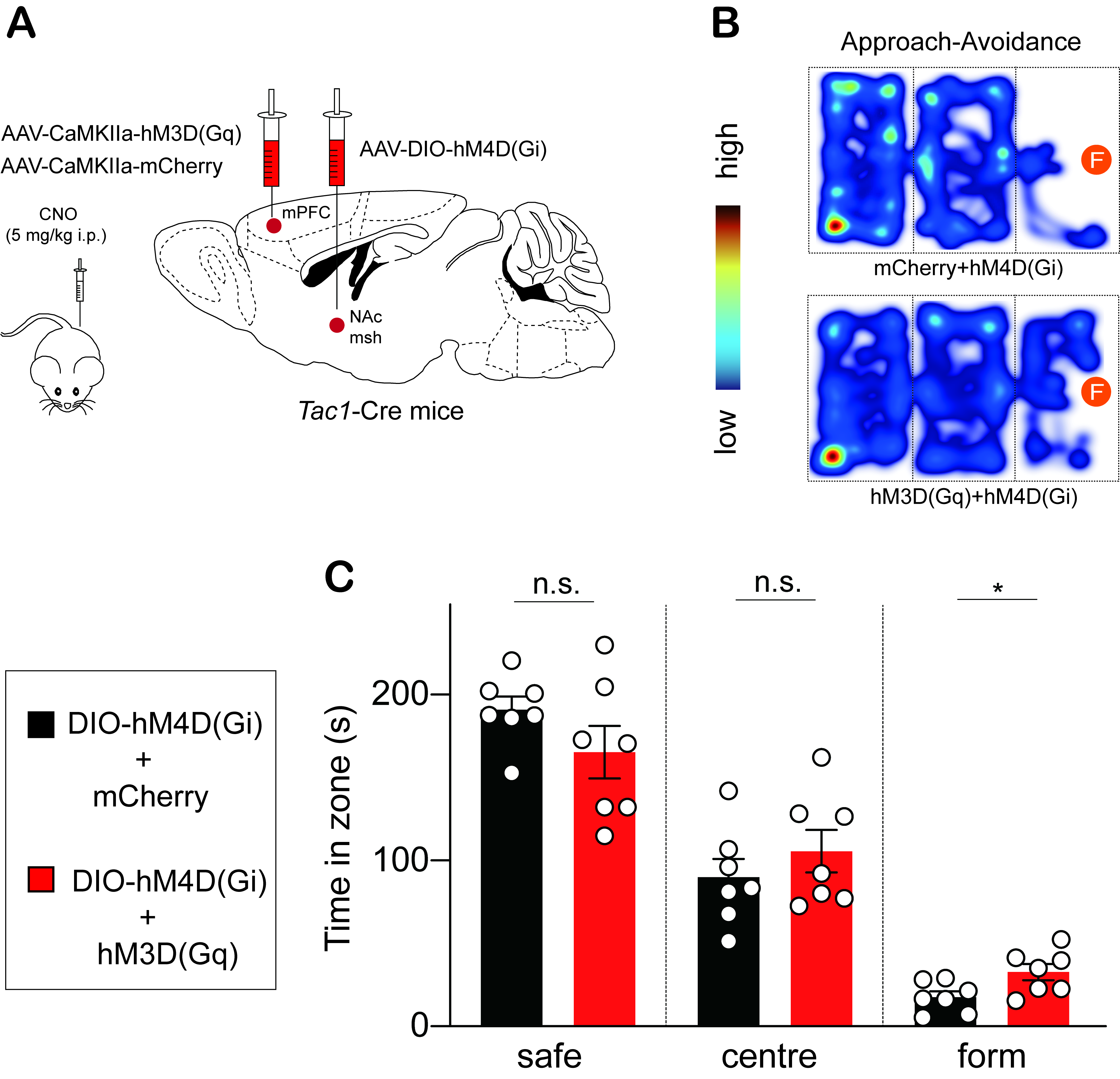

Supplement: Supplementary file 1 [file ijms-24-04346-s001.zip › Figure S11.tif]

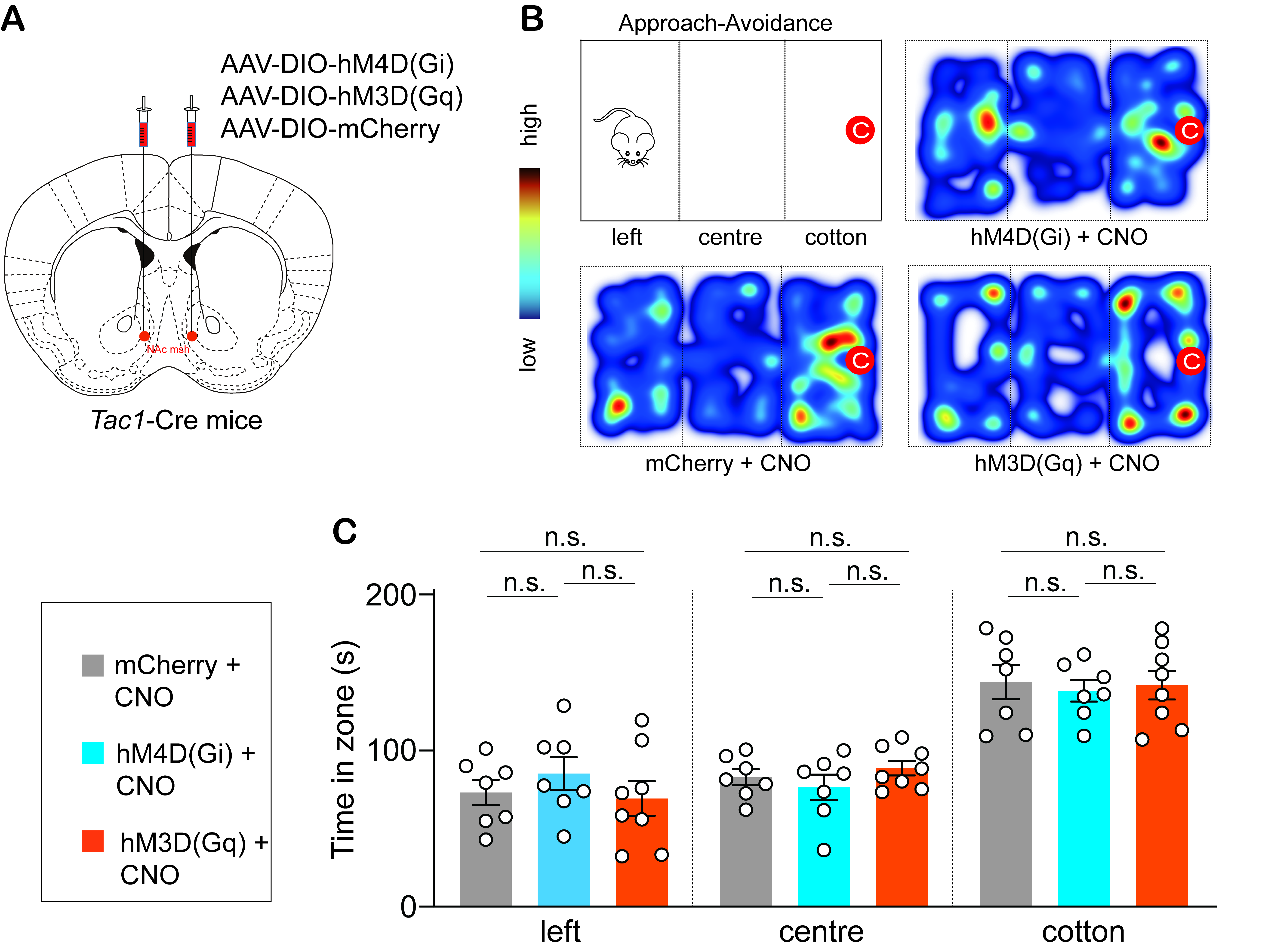

Supplement: Supplementary file 1 [file ijms-24-04346-s001.zip › Figure S2.tif]

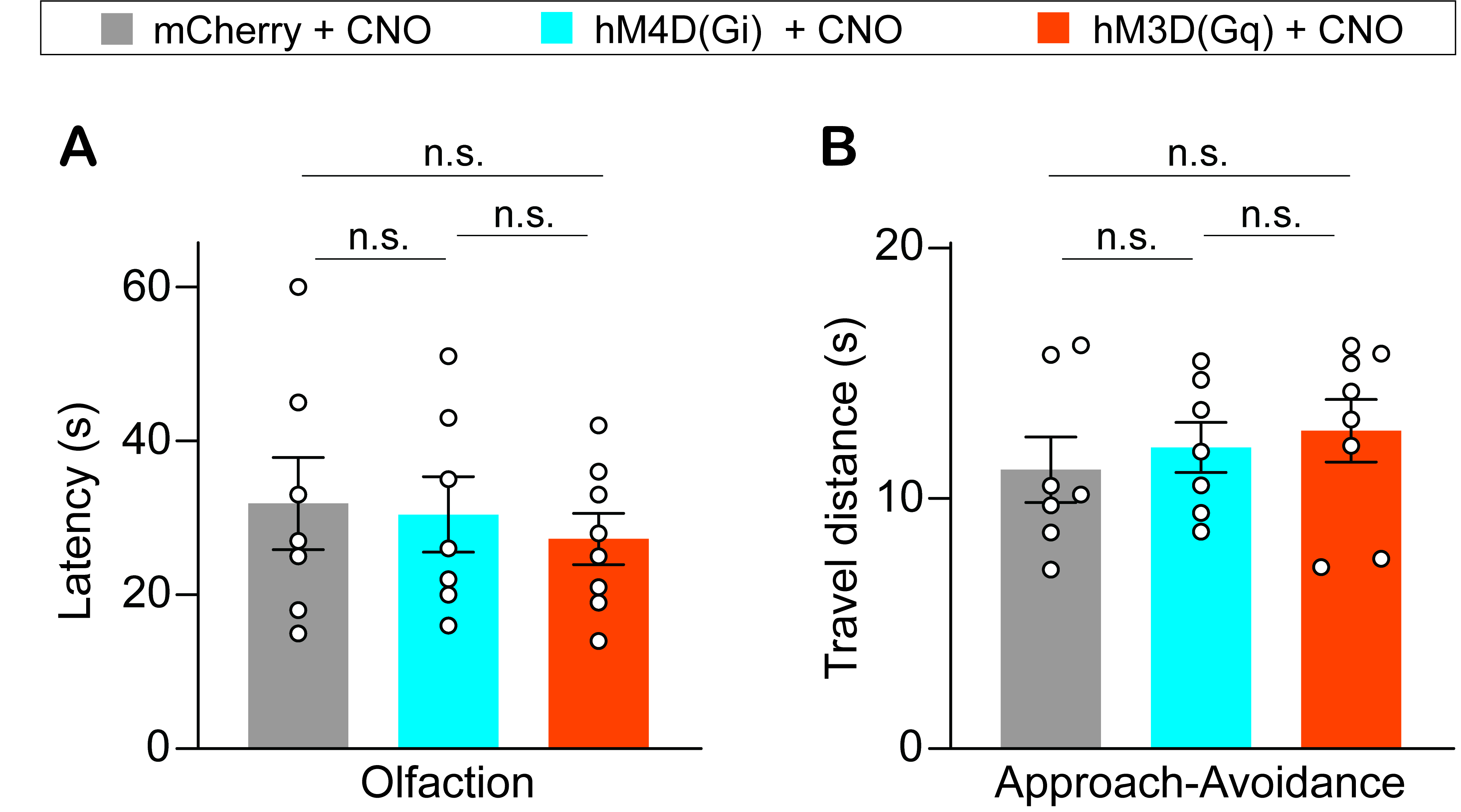

Supplement: Supplementary file 1 [file ijms-24-04346-s001.zip › Figure S3.tif]

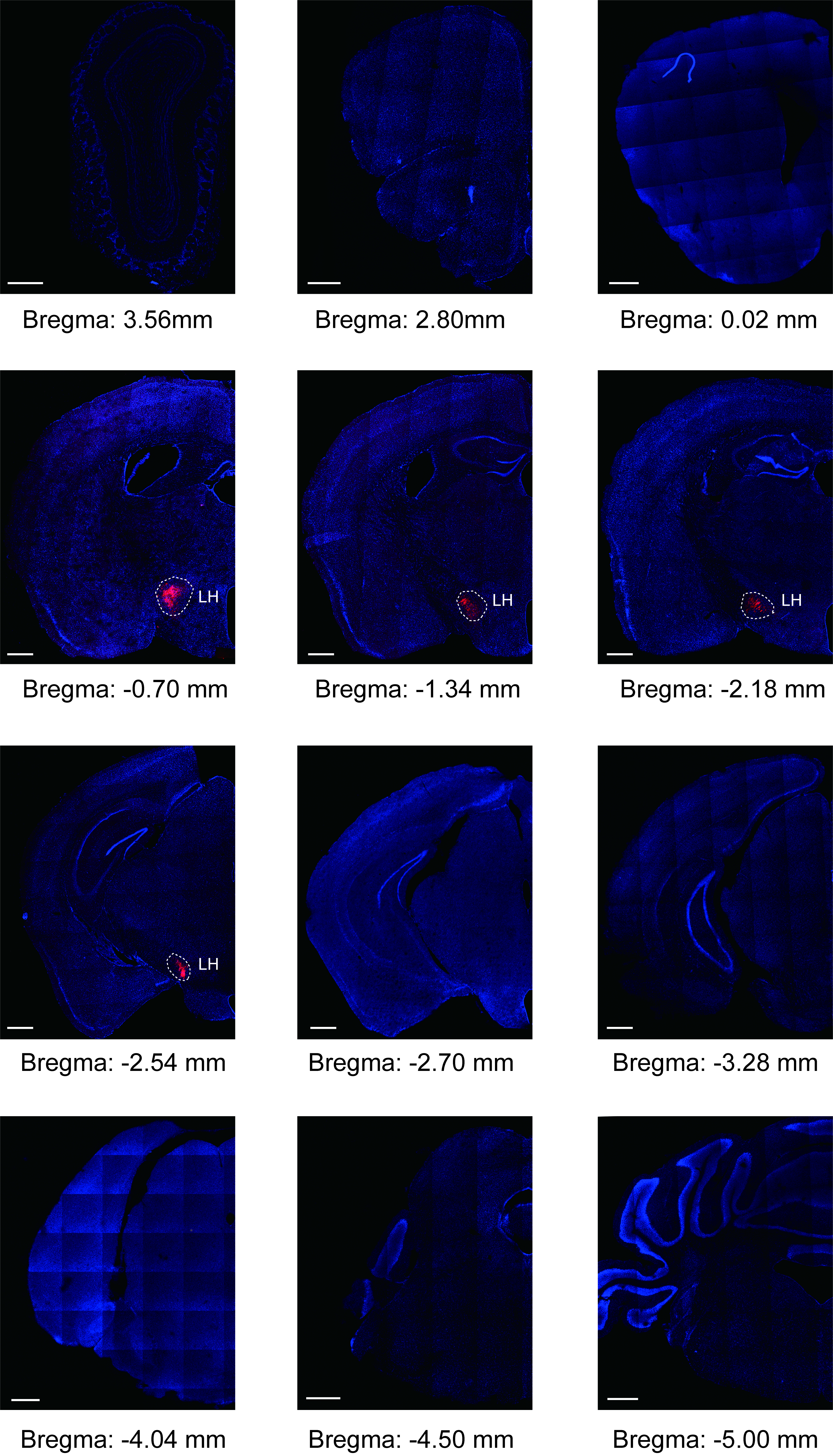

Supplement: Supplementary file 1 [file ijms-24-04346-s001.zip › Figure S4.tif]

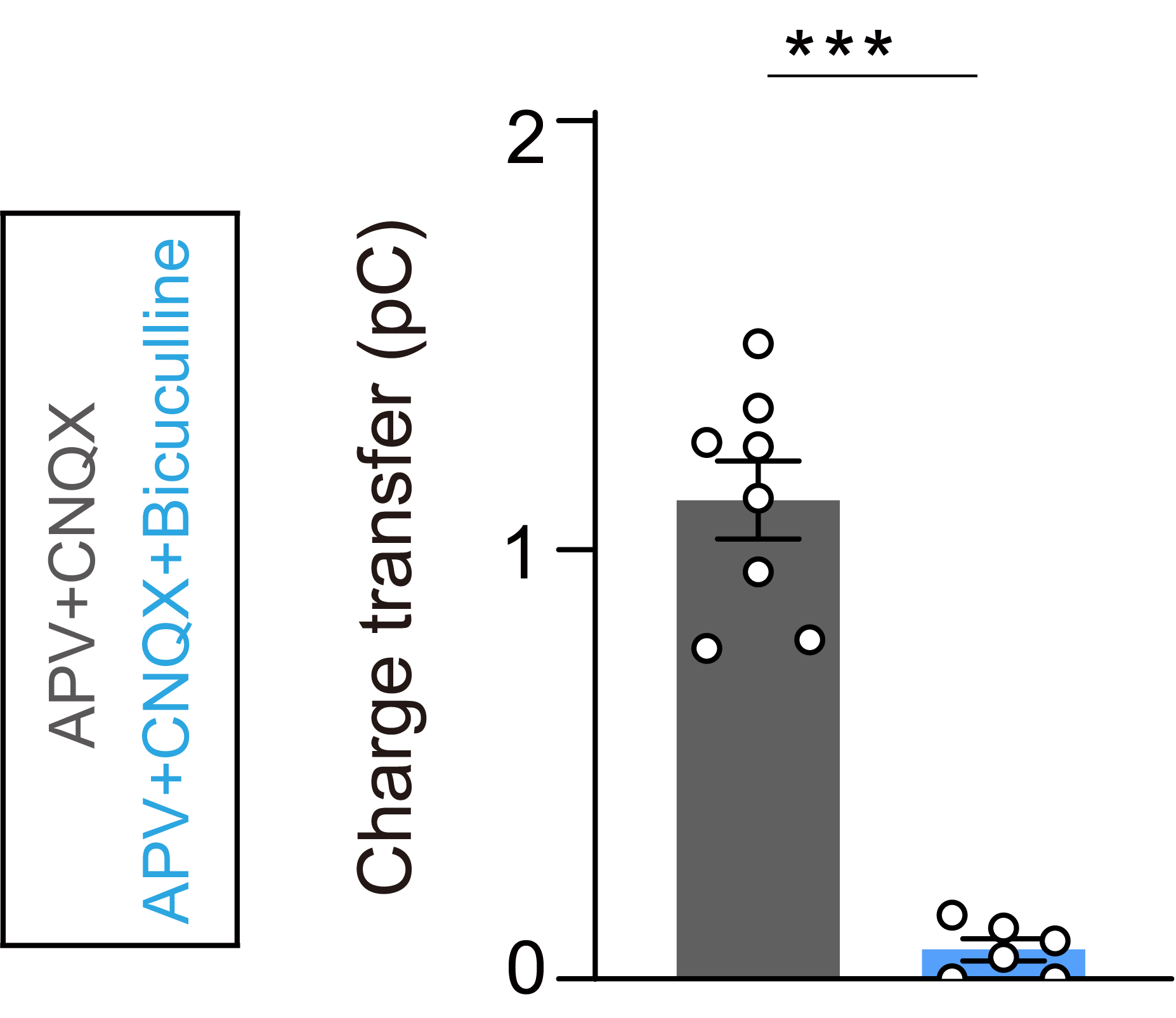

Supplement: Supplementary file 1 [file ijms-24-04346-s001.zip › Figure S5.tif]

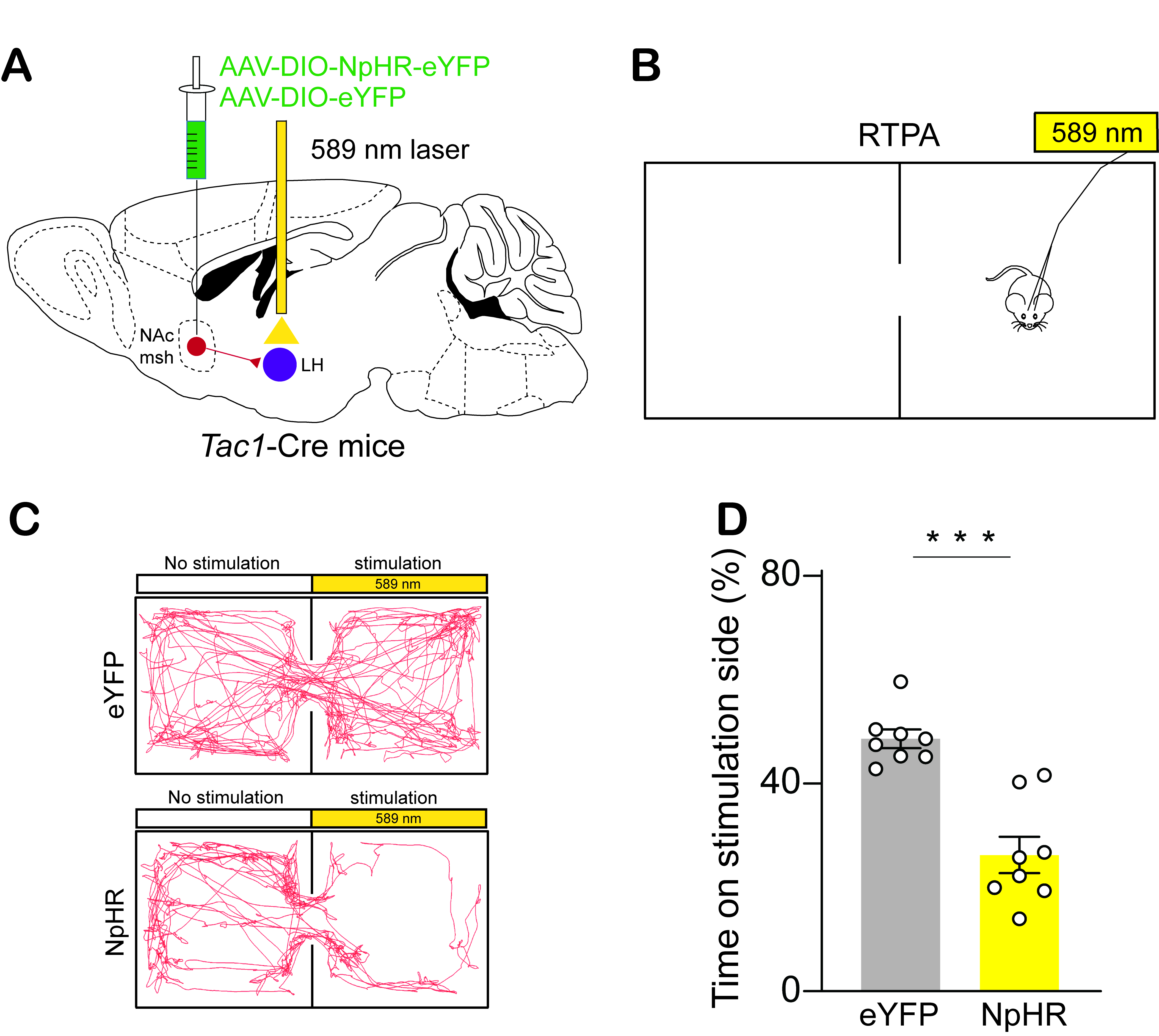

Supplement: Supplementary file 1 [file ijms-24-04346-s001.zip › Figure S6.tif]

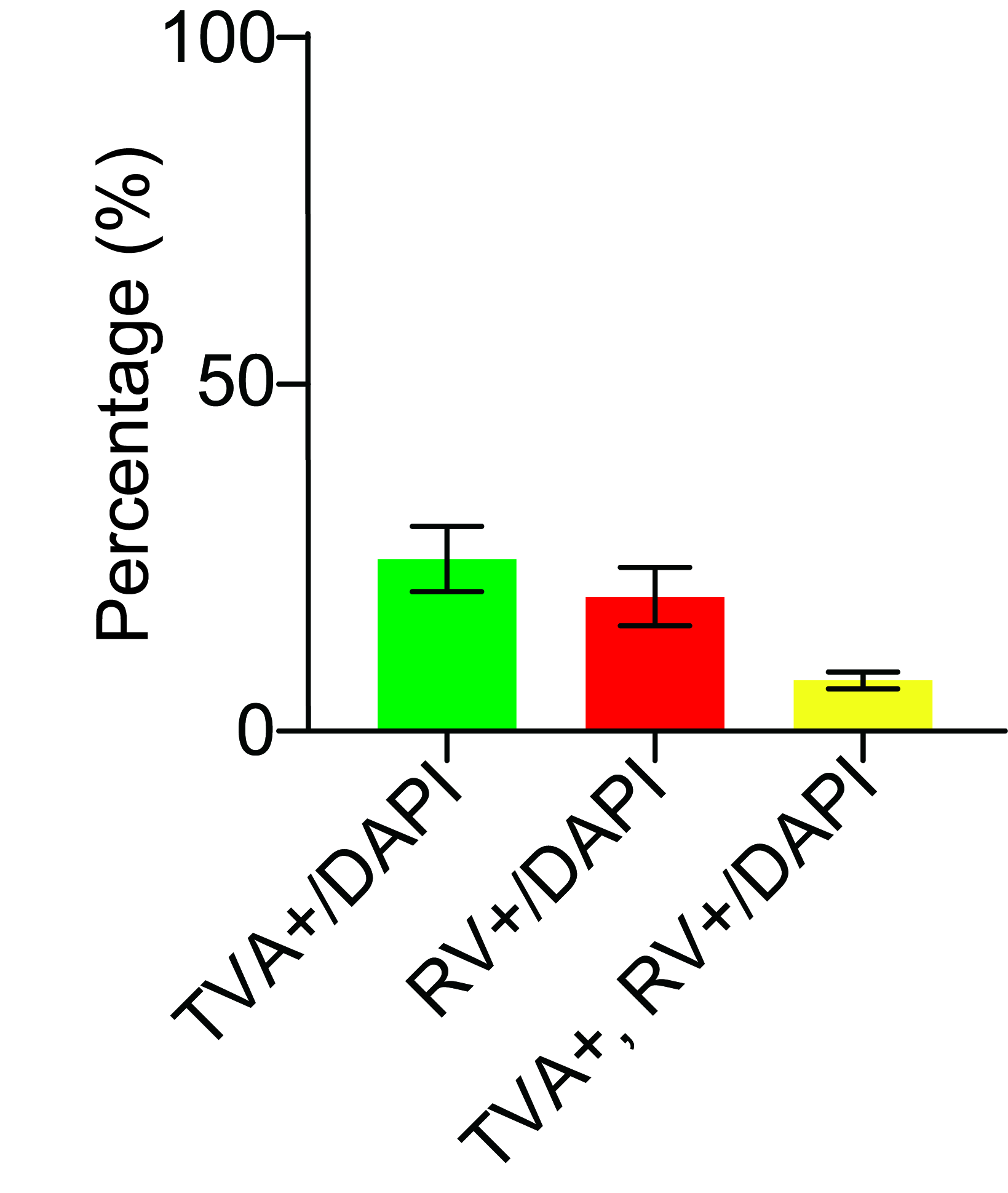

Supplement: Supplementary file 1 [file ijms-24-04346-s001.zip › Figure S7.tif]

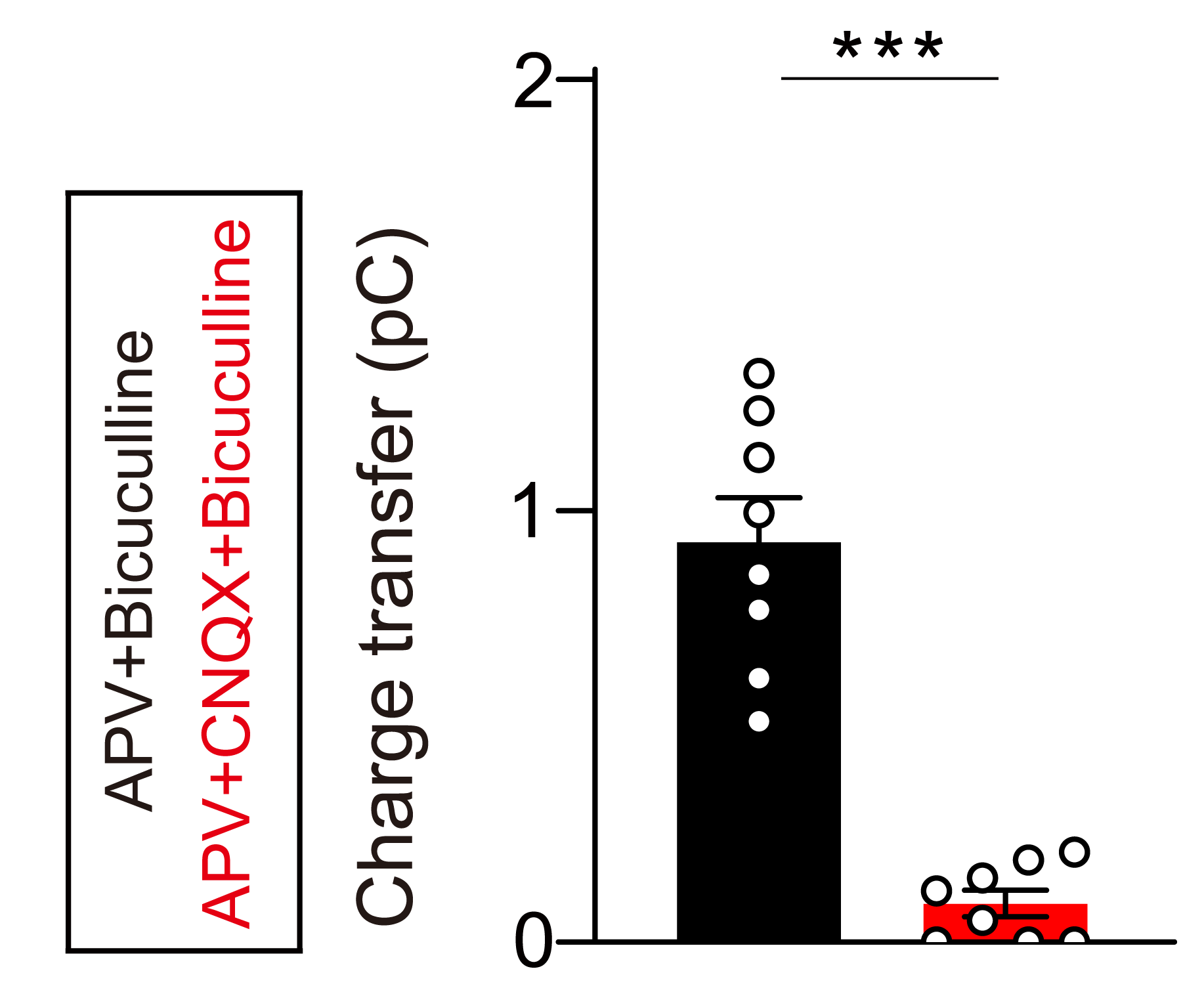

Supplement: Supplementary file 1 [file ijms-24-04346-s001.zip › Figure S8.tif]

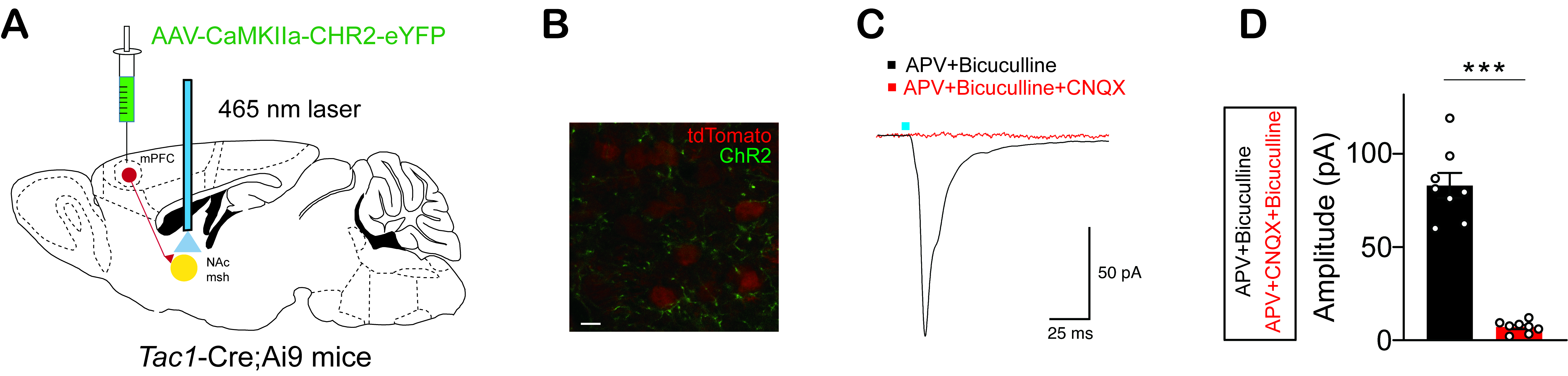

Supplement: Supplementary file 1 [file ijms-24-04346-s001.zip › Figure S9.tif]
